# Predicting peritoneal carcinomatosis of gastric cancer: A simple model to exempt low-risk patients from unnecessary staging laparoscopy

**DOI:** 10.3389/fsurg.2022.916001

**Published:** 2022-07-21

**Authors:** Zhemin Li, Guangmin Guan, Zining Liu, Jiazheng Li, Xiangji Ying, Fei Shan, Ziyu Li

**Affiliations:** Key Laboratory of Carcinogenesis and Translational Research (Ministry of Education/Beijing), Gastrointestinal Cancer Center, Peking University Cancer Hospital & Institute, Beijing, China

**Keywords:** gastric cancer, peritoneal carcinomatosis, risk factor, prediction model, staging laparoscopy

## Abstract

**Background:**

Peritoneal carcinomatosis (PC) of gastric cancer indicates a poor outcome and is mainly diagnosed by staging laparoscopy (SL). This study was designed to develop a risk stratification model based on the number of risk factors to exempt low-risk patients from unnecessary SL.

**Methods:**

This was a retrospective cohort study based on a single institution between January 2015 and December 2019. SL is indicated for patients of advanced locoregional stage, and clinicopathologic characteristics of 535 consecutive patients were included. PC-associated variables were identified by logistic regression analysis. A risk stratification model based on the number of risk factors was constructed, and we defined its predictive value with a receiver operating characteristic (ROC) curve and negative predictive value.

**Results:**

In total, 15.9% of included patients were found to have PC during SL. Borrmann type IV, elevated CA125, and tumour diameter ≥5 cm were independent risk factors of PC. These three factors combined with cT4 were selected as predictive factors, and the number of predictive variables was significantly related to the possibility of PC (2.0%, 12.8%, 20.0%, 54.2%, and 100%, respectively). When the cutoff value is more than one predictive factor, the negative predictive value is 98.0%, with an area under the curve of 0.780. This model could exempt 29.8% of unnecessary SL compared to the indication of the current NCCN guideline.

**Conclusions:**

We constructed a simple model to predict the probability of PC using the number of predictive factors. It is recommended that patients without any of these factors should be exempt from SL.

## Introduction

The incidence of gastric cancer ranks fifth among malignant tumours, and the mortality rate ranks fourth (1). Peritoneal carcinomatosis (PC), including macroscopic carcinomatosis (P1) and positive cytology (CY1), is common among gastric cancer patients and tends to have poor outcomes regardless of the treatment approach (2, 3). Radiographic modalities, such as CT and MRI, have poor sensitivity for diagnosing PC (4–6); thus, staging laparoscopy (SL) combined with peritoneal cytology as a minimally invasive approach has been recommended by therapeutic guidelines, aiming for more accurate M1 staging (7).

However, the indication of SL remains debatable. NCCN guideline recommends that all locoregional patients undergo SL (8), with a 12.5%–20.7% chance of detecting PC (9–12). The relatively low positive rate discourages clinicians from performing it and makes it less cost-effective (13). A population-based study reported that only 8% of patients diagnosed with gastric cancer underwent SL (14). The JGCA guideline recommends only limited patients with large Borrmann III and Borrmann IV tumours and/or Bulky N status require SL (7). However, such a strict indication would raise concerns about omitting PC patients.

Therefore, this study was designed to identify factors related to PC and construct a risk stratification model to predict PC non-invasively and determine who should and should not undergo staging laparoscopy before the treatment.

## Methods

### Study patients

A retrospective analysis of data from patients with gastric cancer admitted to the Gastrointestinal Cancer Center of Peking University Cancer Hospital from January 2015 to December 2019 was performed. As a result, 535 patients were selected according to the inclusion and exclusion criteria.

The inclusion criteria were as follows: (1) pathologically confirmed gastric cancer; (2) clinical stage cT ≥ 2M0 or cN + M0 (no signs of PC by CT or other imaging tools); and (3) received SL. The exclusion criteria were as follows: (1) recurrence of gastric cancer; (2) simultaneous presence of other tumours; (3) received radiotherapy, chemotherapy, immune, or targeted therapy before SL; and (4) unable to extract imaging or clinical data.

### Staging laparoscopy

SL is mandatory for locally advanced stage patients in our center before gastrectomy or applied alone before neoadjuvant therapy, as described in the inclusion criteria. The SL technique was described in a previous study (12). Patients lay supine, and a 10-mm disposable trocar (observation hole) was inserted above or below the umbilicus using the open Hasson method. The abdominal cavity was examined sequentially by a 30° laparoscope. We instilled 500 ml of saline in the abdominal cavity to obtain at least 100 ml of specimens divided into two tubes for centrifugation. Then, one of the tubes was prepared as conventional smears stained with hematoxylin–eosin and as liquid-based cytology samples stained with Papanicolaou stain. A cell pellet was obtained from the other tube and stained with hematoxylin–eosin. Nodules suspected of macroscopic carcinomatosis (P1) during inspection of the abdominal cavity were biopsied. The biopsy results determined the status of macroscopic carcinomatosis (P1 or P0). The cytology status (CY1 or CY0) was based on the peritoneal lavage results, which were performed before gastric cancer resection. Patients with P1 and/or CY1 were defined as PC+; those with P0 and CY0 were defined as PC−.

### Clinical variables

The cTNM stage and Borrmann type were determined based on the enhanced CT images of the abdomen performed at our hospital before SL; the method was consistent with that described by Kim et al. (15). The gross appearance of CT of the tumour was categorized into Borrmann type I–IV: type I, nodular polypoid tumour without ulceration; type II, an ulcerative lesion but distinct borders; type III, an ulcerating tumour with infiltrating ulcer base; and type IV, diffuse thickening of the gastric wall without distinct ulceration. Tumour diameter was defined as the largest diameter in the transverse, sagittal, or coronal position in the same CT. According to the pathology report, Lauren's classification, degree of differentiation, and signet ring cell carcinoma were determined. The levels of plasma tumour markers were determined according to the test results obtained at our hospital before SL.

### Statistical analysis

Associations between clinicopathologic variables and diagnosis of carcinomatosis or positive cytology were examined using chi-square and Fisher's exact tests. Multivariate logistic regression models were used to identify risk factors for PC. Receiver operating characteristic (ROC) curves and negative predictive values were used to determine the diagnostic power of the model. *P* < 0.05 was defined as statistically significant. Internal validation was trained in the form of 10-fold cross-validation over 1,000 iterations using the “caret” package in R (version 4.1.1). All other statistical analyses were conducted using IBM SPSS Statistics (version 26; IBM Corp., NY, USA).

## Results

### Clinical characteristics

The clinical characteristics of 535 patients with locally advanced gastric cancer who had SL are presented in Table 1. A total of 376 patients were males (70.3%), and 159 were females (29.7%). The average age was 59.1 ± 11 years. In total, 61.5% of patients were staged as cT4, while 82.2% were cN+. The numbers of patients with Borrmann I–IV were 16 (3.0%), 70 (13.1%), 404 (75.5%), and 45 (8.4%), respectively. The average tumour diameter was 47.8 ± 22.5 mm. SL showed PC in 85 patients. Among them, 11 patients (13.0%) had P1 only, 45 patients (53.0%) had CY1 only, and 29 (34.1%) patient had both P1 and CY1.

**Table 1:** Clinicopathologic features of patients and results of univariate analysis and multivariate analysis.

Variables	PC-(P0CY0)	PC+(P1 and/or CY1)	Missing value (*n*%)	Univariate analysis		Multivariate analysis	
				*P* value	OR (95% CI)	*P* value	OR (95% CI)
Gender			NA	*P* = 0.028	1.733 (1.072–2.803)		
Male	325	51					
Female	125	34					
Age			NA	*P* = 0.062	1.136 (0.698–1.848)		
<65 years	304	55					
≥65 years	146	30					
Clinical T stage			NA	*P* < 0.0001	3.441 (1.911–6.197)	0.180	1.664 (0.790–3.502)
cT1-3	191	15					
cT4	259	70					
Clinical N stage			NA	*P* < 0.0001	2.759 (1.685–4.517)	0.295	1.436 (0.730–2.823)
cN0-1	253	27					
cN2-3	197	58					
Borrmann type			NA	*P* < 0.0001	9.996 (5.213–19.17)	0.000	4.876 (2.146–11.078)
I–III	431	59					
IV	19	26					
Tumor diameter			NA	*P* < 0.0001	5.670 (3.360–9.570)	0.004	2.762 (1.380–5.525)
<5 cm	299	22					
≥5 cm	151	63					
Lauren type			28 (5.2%)	*P *< 0.0001	2.636 (1.612–4.310)	0.135	1.733 (0.843–3.564)
Non-diffused	311	39					
Diffused	118	39					
Differentiation			53 (9.9%)	*P* = 0.001	2.824 (1.442–5.532)	0.309	1.544 (0.669–3.564)
Moderate-well	133	11					
Low	274	64					
Signet-ring cell			8 (1.5%)	*P* = 0.006	1.994 (1.235–3.219)	0.329	1.426 (0.700–2.908)
No	323	49					
Yes	119	36					
CA125			32 (6.0%)	*P *< 0.0001	5.414 (2.580–11.365)	0.003	4.277 (1.618–11.305)
<35 U/ml	405	66					
≥35 U/ml	17	15					
CEA			13 (2.4%)	*P* = 0.645	1.156 (0.644–2.076)		
<5 ng/ml	359	66					
≥5 ng/ml	80	17					
AFP			42 (7.9%)	*P* = 1.000	1.015 (0.409–2.521)		
<7 ng/ml	383	73					
≥7 ng/ml	31	6					
CA199			36 (6.7%)	*P* = 0.061	1.805 (0.997–3.267)		
<37 U/ml	361	60					
≥37 U/ml	60	18					
CA724			14 (2.6%)	*P* = 0.097	2.419 (0.868–5.321)		
<35.4 U/ml	420	76					
≥35.4 U/ml	18	7					

PC, peritoneal carcinomatosis; OR, odds ratio; CI, confidence interval.

### Results of univariate analysis and multivariate analysis

The relationships between the clinical factors and PC are shown in Table 1. In the univariate analysis, cT4, N2–3, tumour diameter ≥5 cm, Borrmann IV, Lauren diffused carcinoma, poorly differentiated carcinoma, signet ring cell carcinoma, and plasma CA125 ≥ 35 U/ml were significantly related to PC.

In the multivariate analysis of these factors among 444 patients, Borrmann type IV (OR 4.88; 95% CI, 2.15 to 11.08; *P* < 0.0001), CA125 ≥ 35 U/ml (OR 4.28; 95% CI, 1.62–11.30; *P* = 0.003), and tumour diameter ≥5 cm (OR 2.76; 95% CI, 1.38–5.52; *P* = 0.004) were independent risk factors of PC.

### Predictive model and diagnostic performance

All three independent risk factors identified by logistic regression analysis, combined with cT4, were selected as predictive factors of PC. As shown in Table 2, the proportions of patients with PC who had 0–4 risk factors were 2.0% (3/150), 12.8% (23/180), 20.0% (24/120), 54.2% (26/48), and 100% (5/5), respectively.

**Table 2:** Proportion of patients with PC in different groups.

Number of predictive factors	Patients with PC/total patients	Proportion of PC (%)
0	3/150	2.0
1	23/180	12.8
2	24/120	20.0
3	26/48	54.2
4	5/5	100

PC, peritoneal carcinomatosis.

The AUC value of the model was 0.780 with a 95% CI of 0.728–0.831 (Figure 1). The average AUC based on 1,000 tenfold validations was 0.784 with a 95% CI of 0.616–0.924 (Figure 2). The diagnostic sensitivity, specificity, positive predictive value, and negative predictive value of each cutoff value are presented in Table 3. Notably, the sensitivity and negative predictive values are 96.3% and 98.0%, respectively, when staging laparoscopy is to have at least one predictive factor.

**Table 3:** Sensitivity, specificity, positive predictive value, and negative predictive value of the model with different cutoff values.

Cutoff value	Sensitivity (%)	Specificity (%)	Positive predictive value (%)	Negative predictive value (%)
1^a^	96.3	34.8	22.1	98.0
2	68.0	72.0	31.8	92.1
3	38.3	94.7	58.5	88.9
4	6.2	100	100	84.7

^a^Patients with one or more risk factors requiring staging laparoscopy.

### Comparison with the indication for staging laparoscopy in guidelines

In addition, we compare the diagnostic performance of different indications for SL by using two parameters: yields of staging laparoscopy and omitted PC from patients exempted from staging laparoscopy (Table 4). According to the NCCN guideline, which was an indication for SL in our center, all patients were recommended to have SL, of which the positive rate was 16%. Although, according to the JGCA guideline, most patients were exempted from SL, 53 of these 469 patients (11.3%) had PC. According to our study, 150 patients without risk factors, accounting for 29.8% of all patients, could be exempt from staging laparoscopy, and only 3 (2%) patients with PC would be missed.

**Table 4:** Results of staging laparoscopy according to different guidelines.

Guideline	Indication for staging laparoscopy	Patients requiring laparoscopy and proportion of PC	Patients free of laparoscopy and proportion of PC
JGCA 5th	Borrmann IV or large Borrmann III or bulky lymph nodes^a^	33/66 (50.0%)	53/469 (11.3%)
NCCN 2021	T1b-T4 or N+ and cM0	86/535 (16.0%)	0
CSCO 2021	Peritoneal metastasis suspected by CT; T3-4/N+ patients ready for neoadjuvant therapy	86/521 (16.5%)	0/14 (0%)
Model in our study	≥1 predictive factor	78/353 (22.1%)	3/150 (2.0%)

^a^Information about Bulky lymph nodes could not be obtained from CT, so only Borrmann type was included in the analysis.

## Discussion

This study proposed a risk stratification model of peritoneal carcinomatosis using four widely applied factors: clinical T stage, Borrmann type, tumour diameter, and CA125. The percentages of PC in patients with 0–4 risk factors were 2.0%, 12.8%, 20.0%, 54.2%, and 100%, respectively. The model could spare 29.8% of patients from SL compared with the current NCCN guideline, improving medical efficiency and reducing medical costs for patients without any risk factors.

Guidelines provide different indications for SL in patients with advanced gastric cancer. Japanese investigators apply indication for SL the same as neoadjuvant chemotherapy: gastric cancer with “bulky N” status and Borrmann type IV and large type III gastric cancer. The yield of this indication is reported to be 53.4%–56.8% (16), consistent with the result (50%) of our research. However, strict indications might omit potential PC patients from accurate staging. In our study, patients who do not meet the indication of JGCA guidelines still have an 11.3% chance of PC.

On the other hand, western countries chose indication for SL as “resectable GC and EGJ without definite distant metastases” (8), but a population-based study reported only 8% of patients diagnosed with gastric cancer underwent SL (14). Furthermore, too broad an indication introduces the problems of “many negative SL” and “cost-effectiveness” (17). Thus, patient selection for SL is still controversial, and the population that does not need SL must be carefully inspected.

Studies attempted to develop prediction models by constructing nomograms or summarizing high-risk factors by multivariate analysis. For example, Yang et al. constructed a nomogram based on the tumour size, degree of differentiation, and Glasgow prognosis score (18). Similarly, Zhao et al. used five factors, including weight loss and level of H5N5 (a kind of N-glycan), for his nomogram (19). Dong et al. used the Lauren classification and radiomics features from abdominal CT imaging to build a nomogram (20). A nomogram might result in a high AUC value, but it requires detailed calculations and often involve factors not commonly used in the clinical scenario. Thus, we had no intention of building a new one in this study. Studies also used the number of risk factors as an indication for SL; for example, Hu et al. used tumour size, cT4b, and Borrmann type III or IV as their risk factors (21). However, the missing CA125 might weaken their conclusion and lacks direct comparison with the current guideline.

In our study, clinical stage T4, Borrmann type IV, tumour diameter ≥5 cm, and CA125 ≥ 35 U/ml were selected as risk factors, which have a theoretical basis. The cT4 stage complies with studies before and with Paget’s “seed and soil” hypothesis, which suggests that as the primary tumour penetrates the serosa, more cancer cells will be shed into the abdominal cavity. Thus, the frequency of PC will also increase (22). Borrmann type IV cancer is often present with the peritoneal disease and selected as one of the indications for SL in Japan; the incidence of PC in B-IV cancer was reported to be as high as 58%–78% in Japanese studies (23, 24). Tumour diameter is not recommended as indicated in western guidelines, but multiple studies have shown that tumour diameter was an independent risk factor for PC (11, 21, 25–27). Previous studies less mentioned CA0, maybe because the antigen CA125 is produced by normal epithelia and not tumour-specific (28, 29). However, our data indicate that nearly half of patients with elevated CA125 were confirmed to have PC in the following SL, which was reported by previous articles (28, 30, 31). Up to 58% of patients with Borrmann IV had PC; the proportion also reached 47% in patients with elevated CA125. These four factors are significantly related to PC, while common staging modalities can obtain them.

We also made a comparison with other combinations of risk factors. Removal of any of the elements from the model resulted in a significant decrease in its diagnostic efficacy, and the addition of other factors did not increase its diagnostic effectiveness. Comparisons between the different models are detailed in the Supplementary Materials, including a comparison with the model consisting of the three factors statistically associated with PC in the multivariate analysis. Unlike the current guideline, the N stage is not included in our model because the statistical correlation in our study is not significant and the N stage fails to show independent correlations in multiple previous studies (12, 32–35). As shown in Table 4, the negative predictive value was 98.0%, with a cutoff value of 1. However, the results for the other cutoff values were not satisfactory.

There are some limitations to the study. First, we did not separate macroscopic carcinomatosis from positive cytology in the analysis. Second, there were missing values in the multivariate analysis, and the efficiency of the predictive model was also not verified by external data. Finally, the staging laparoscopy strategy for patients with one or more risk factors requires further investigation and clarification.

In conclusion, we propose a model consisting of four factors, including Borrmann type IV, tumour diameter ≥5 cm, CA125 ≥ 35 U/ml, and cT4, for predicting PC in patients with advanced gastric cancer. We recommend that patients without any of these risk factors could be exempt from staging laparoscopy before treatment and the others, especially ones with Borrmann IV gastric cancer and/or elevated CA125, should undergo staging laparoscopy.

## Data Availability

The raw data supporting the conclusions of this article will be made available by the authors, without undue reservation.
