# Supplementary figures and images for: Predicting peritoneal carcinomatosis of gastric cancer: A simple model to exempt low-risk patients from unnecessary staging laparoscopy
Source: Front Surg. 2022 Jul 21;9:916001. doi: 10.3389/fsurg.2022.916001 (PMC9349356; doi:10.3389/fsurg.2022.916001)

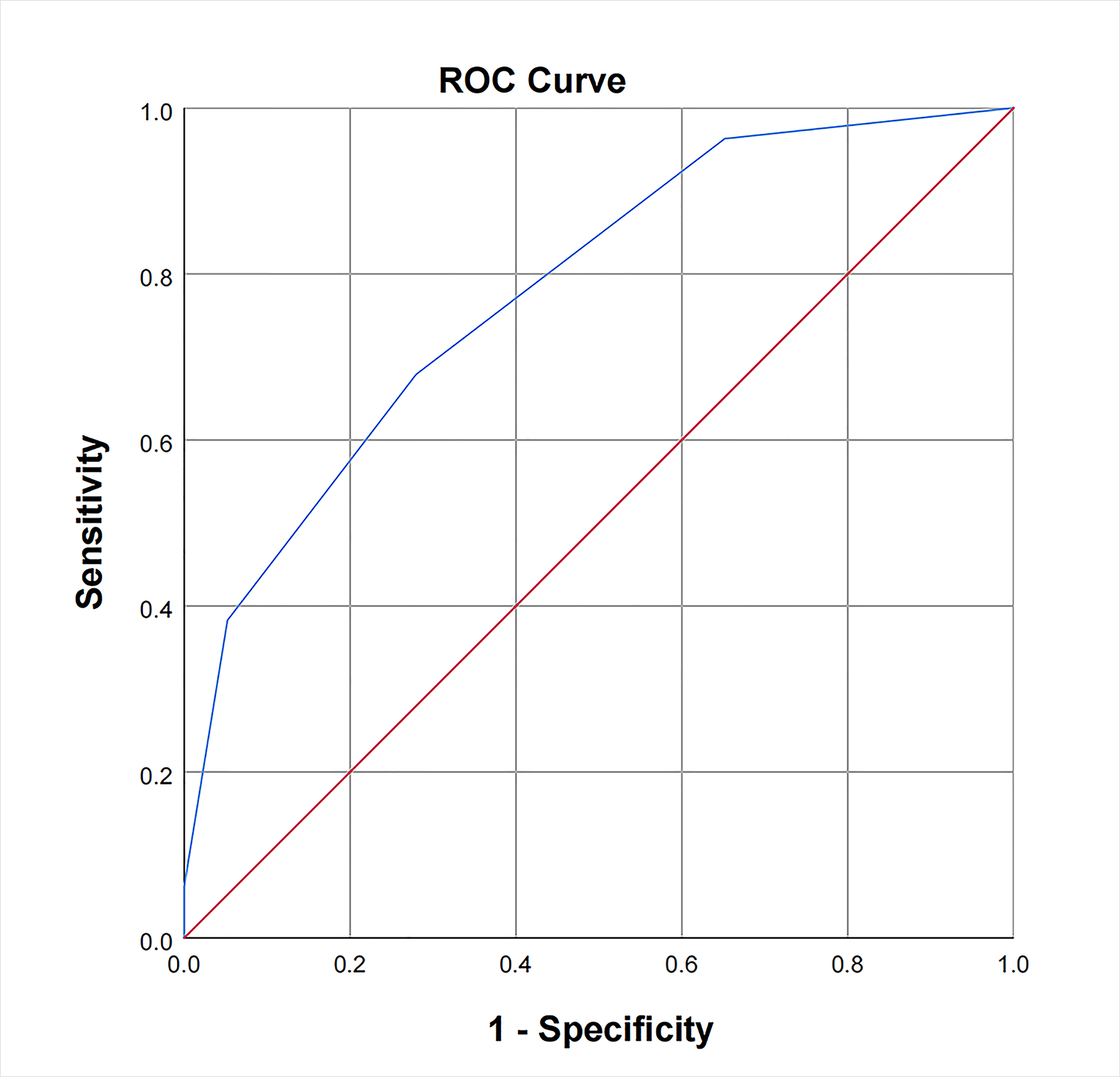

Supplement: Supplementary file 1 [file Figure_1_v1.tif]

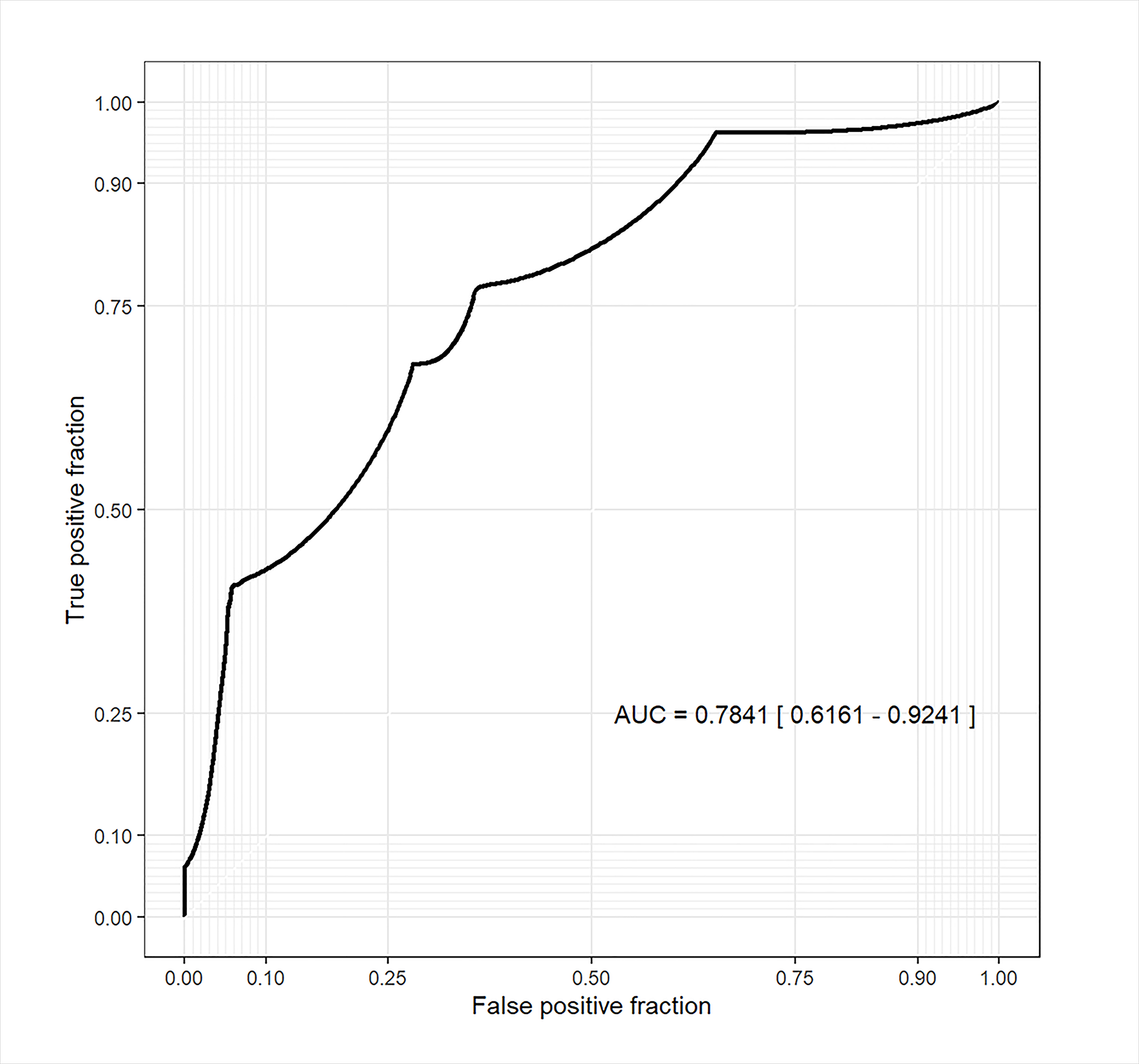

Supplement: Supplementary file 2 [file Figure_2_v1.tif]
